# Dysbiosis of Gut Microbiota and Short-Chain Fatty Acids in Encephalitis: A Chinese Pilot Study

**DOI:** 10.3389/fimmu.2020.01994

**Published:** 2020-08-20

**Authors:** Ruoting Xu, Chuhong Tan, Yan He, Qiheng Wu, Huidi Wang, Jia Yin

**Affiliations:** ^1^Department of Neurology, The First Affiliated Hospital of Wenzhou Medical University, Wenzhou, China; ^2^Department of Critical Care Medicine, Nanfang Hospital, Southern Medical University, Guangzhou, China; ^3^Microbiome Medicine Center, Division of Laboratory Medicine, Zhujiang Hospital, Southern Medical University, Guangzhou, China; ^4^Department of Neurology, Nanfang Hospital, Southern Medical University, Guangzhou, China

**Keywords:** gut microbiome, dysbiosis, encephalitis, short-chain fatty acids, intestinal barrier

## Abstract

**Background:**

Encephalitis, the inflammation of the brain, may be caused by an infection or an autoimmune reaction. However, few researches were focused on the gut microbiome characteristics in encephalitis patients.

**Methods:**

A prospective observational study was conducted in an academic hospital in Guangzhou from February 2017 to February 2018. Patients with encephalitis were recruited. Fecal and serum samples were collected at admission. Healthy volunteers were enrolled from a community. Disease severity scores were recorded by specialized physicians, including Glasgow Coma Scale (GCS), Sequential Organ Failure Assessment (SOFA), and Acute Physiology and Chronic Health Evaluation-II (APACHE-II). 16S rRNA sequence was performed to analyze the gut microbiome, then the α-diversities and β-diversities were estimated. Short-chain fatty acids (SCFAs) were extracted from fecal samples and determined by gas chromatography-mass spectrometry. Serum D-lactate (D-LA), intestinal fatty acid-binding protein (iFABP), lipopolysaccharide (LPS), and lipopolysaccharide-binding protein (LBP) were measured by enzyme-linked immunosorbent assay (ELISA). The associations among microbial indexes and clinical parameters were evaluated by Spearman correlation analysis.

**Results:**

In total, twenty-eight patients were recruited for analysis (median age 46 years; 82.1% male; median GCS 6.5; median SOFA 6.5; median APACHE-II 14.5). Twenty-eight age- and sex-matched healthy subjects were selected as controls. The β-diversities between patients and healthy subjects were significantly different. The α-diversities did not show significant differences between these two groups. In the patient group, the abundances of *Bacteroidetes*, *Proteobacteria*, and *Bacilli* were significantly enriched. Accordingly, fecal SCFA levels were decreased in the patient group, whereas serum D-LA, iFABP, LPS, and LBP levels were increased compared with those in healthy subjects. Correlation analyses showed that disease severity had positive correlations with *Proteobacteria* and *Akkermansia* but negative correlations with *Firmicutes*, *Clostridia*, and *Ruminococcaceae* abundances. The cerebrospinal fluid albumin-to-serum albumin ratio (CSAR) was positively related to the α-diversity but negatively correlated with the fecal butyrate concentration.

**Conclusion:**

Gut microbiota disruption was observed in encephalitis patients, which manifested as pathogen dominance and health-promoting commensal depletion. Disease severity and brain damage may have associations with the gut microbiota or its metabolites. The causal relationship should be further explored in future studies.

## Introduction

Encephalitis, an acute inflammation of the central nervous system (CNS) associated with neurologic dysfunction, is a public health concern worldwide because of its high mortality and neurological sequelae rates (1). The reported incidence of acute encephalitis varies worldwide but is generally estimated to be 1.7–7.4 cases per 100,000 person-years (2). Causes of encephalitis include viruses, bacteria, fungi, and parasites (2). Other causes include autoimmune diseases and certain medications (3). In many cases, the etiology remains unknown (4). Diagnosis is typically based on symptoms and supported by blood tests, medical imaging, and analysis of cerebrospinal fluid (5).

Some encephalitis may lead to irreparable brain damage. Symptoms common to most types of encephalitis are headache, fever, altered mentation, seizures, and focal neurological signs (6). Patients require intensive medical care, with continuous monitoring of their heart and respiratory functions and management of their fluid and electrolyte balances (7). Although the prognosis varies among different patients, the mortality can be as high as 70%. In 2015, encephalitis was estimated to have affected 4.3 million people and resulted in 150,000 deaths worldwide (8, 9). Treatments for encephalitis remain poor and still suffer from serious shortcomings in most intensive care units.

Current research efforts include gaining a better understanding of how the systemic immune system responds to inflammation in the brain. A better understanding of the gut-microbiota-brain axis involved in the protection and disruption of the blood-brain barrier could lead to the development of new treatments for neuroinflammatory diseases. Previous studies have demonstrated intestinal flora dysbiosis in neurological diseases (10), e.g., stroke (11, 12), multiple sclerosis (13, 14), and neuromyelitis optica spectrum disorders (15). Despite extensive microbiome investigations in CNS diseases, few studies have focused on the features of the intestinal flora in patients with encephalitis. Therefore, investigations into the gut microbiome of encephalitis patients using culture-independent techniques to confirm and characterize these features are urgently needed.

In the present pilot study, 16S rRNA gene sequence analysis was used to describe the phylogenetic composition of the fecal microbiota in a cohort of encephalitis patients and compare the results with those for healthy subjects. Specifically, the concentrations of short-chain fatty acids (SCFAs) in fecal samples and levels of gut permeability biomarkers in serum samples were quantitatively detected. In addition, possibilities to correlate microbiota-associated markers with clinical parameters were also explored.

## Materials and Methods

### Subject Enrollment and Sample Collection

This study was a prospective observational cohort study conducted in the neurological intensive care unit (neuroICU) of an urban academic tertiary referral hospital in Guangzhou for 1 year (staged start between February 2017 and February 2018). Patients were recruited based on the following inclusion criteria: (1) diagnosed with encephalitis by specialized physicians according to definitions from a research study published in Lancet Infect Dis (Supplementary Table S1) (2); (2) admitted to the neuroICU with a Glasgow Coma Scale (GCS) < 11; and (3) had an expected length of intensive care unit (ICU) stay (IOS) of >48 h. Disease severity scores were recorded, including the GCS, Acute Physiology and Chronic Health Evaluation-II (APACHE-II), and Sequential Organ Failure Assessment (SOFA) scores at admission. The GCS is a neurological scale which aims to give a reliable and objective way of recording the state of a person’s consciousness. Patients with low GCS scores have worse brain injury. The SOFA score is used to track a person’s status during the stay in an ICU to determine the extent of a person’s organ function or rate of failure. The APACHE-II score is a severity-of-disease classification system, one of several ICU scoring systems. Patients with high levels of SOFA and APACHE-II scores might have worse prognosis. Self-reported healthy volunteers were recruited from the Bureau of Reclamation in Guangzhou between November 2016 and January 2017. The exclusion criteria for all the subjects were as follows: (1) aged less than 18 years old or more than 80 years old; (2) had used antibiotics, prebiotics or probiotics in the last year prior to blood and feces collection; (3) had gastrointestinal disease, (4) had malignant cancer, or (5) were pregnant. Fecal samples and fasting blood samples were obtained from the patients within 72 h after admission and were collected once from individuals in the control group. Written informed consent was obtained from all healthy subjects and patients or their legal representatives. Ethical approval for both the patients and healthy subjects was received from the Medical Ethics Committee of Nanfang Hospital (No. NFEC-2018-034), and all studies were conducted in accordance with the Declaration of Helsinki.

### Biochemical Tests and Blood-Brain Barrier Biomarkers

Routine blood samples for biochemical tests were obtained within 24 h of hospital admission. All examinations were strictly performed at the laboratory in the hospital. Data were recorded from the hospital information system, including white blood cell count (WBC, ×10^9^/L), neutrophil count (NEU, ×10^9^/L), red blood cell count (RBC, ×10^12^/L), hemoglobin level (HGB, g/L), platelet count (PLT, ×10^9^/L), alanine aminotransferase level (ALT, U/L), total bilirubin level (Tbil, μmol/L), total protein level (TP, g/L), albumin level (ALB, g/L), serum chlorine level (Cl, mmol/L), serum potassium level (K,mmol/L), blood urea nitrogen level (BUN, mmol/L), serum creatinine level (SCr, μmol/L), C-reactive protein level (CRP, mg/L), procalcitonin level (PCT, ng/mL), D-dimer level (DD, mg/L), brain natriuretic peptide level (BNP, pg/mL), neuron-specific enolase level (NSE, ng/mL), and S100 calcium-binding protein B level (S100B, μg/L). Lumbar punctures were performed in patients for clinical reasons within 72 h, and cerebrospinal fluid was immediately sent to the hospital laboratory for examination. Cerebrospinal fluid albumin (CSFA, mg/L) was subsequently recorded. The cerebrospinal fluid albumin-to-serum albumin ratio (CSAR) was used to evaluate blood-brain barrier permeability, as described previously (16).

### Bacterial DNA Extraction and Amplification of 16S rRNA Genes

Fresh stool samples were stored at −80°C within 3 h after voiding, and 0.2 g of each was aliquoted for DNA extraction. Bacterial DNA was extracted with a magnetic bead-based stool DNA extraction kit (Shenzhen Bioeasy Biotechnology Co., Ltd., China) according to the manufacturer’s instructions (17). Using a LightCycler 480 II real-time fluorescence quantitative PCR system (Roche Diagnostics Ltd., Switzerland), the V4 region of the bacterial 16S rRNA gene was amplified by quantitative real-time polymerase chain reaction (q RT-PCR) with the bar-coded primers V4F (5′-GTGTGYCAGCMGCCGCGGTAA-3′) and V4R (5′-CCGGACTACNVGGGTWTCTAAT-3′). Samples that produced a visible product 290–310 bp in length were used for further experiments. The PCR products were mixed in equimolar ratios and purified by an EZNA Gel Extraction Kit (Omega, United States). Finally, 16S rRNA sequencing was conducted on an Illumina HiSeq 2500 platform, and 250-bp paired-end reads were generated.

### Sequencing and Microbial Analysis

Sequences longer than 200 bp were trimmed to 200 bp, and those shorter than 200 bp were removed. Depending on the overlap, we then used SeqPrep to merge the paired-end sequences and assessed the quality of the results using open-source software Quantitative Insights into Microbial Ecology (QIIME, version 1.9.1) (18). The quality of the sequences were checked in QIIME. The sequences with Phred score ≥ Q20 were considered as qualified sequences. Then, we split FASTA files based on the paired-end barcode information, which matched 100% between the barcode and the primer remained more than 200 bp after removal of the barcode and primer. After that, we removed chimeras, performed reference-based operational taxonomic unit (OTU) clustering, and finally generated a BIOM file. All samples were normalized to 7000 sequences to avoid possible errors due to the use of different sequencing depths. The α-diversity (the complexity within a community) was estimated by four indexes and calculated by QIIME (18): (a) Chao1; (b) observed species; (c) Shannon; and d) phylogenetic diversity (PD)-whole tree. The β-diversity (difference between microbial communities) was analyzed using the Bray-Curtis distance and unweighted UniFrac distance (19, 20). To determine the significantly different taxa between two groups, linear discriminant analysis (LDA) coupled with effect size measurement (LEfSe) was performed using an online utility^1^ (21). Significantly different bacteria with LDA scores ≥ 3.5 were diagrammed on cladogram. Phylogenetic Investigation of Communities by Reconstruction of Unobserved States (PICRUSt) algorithm was performed in QIIME to predict the functional profiles of the bacterial metagenomes (Kyoto Encyclopedia of Genes and Genomes, KEGG) in the two groups based on the relative abundance of individual OTUs.

### Fecal Short-Chain Fatty Acid Detection

Fecal samples for SCFA analysis were frozen at −80°C within 3 h of voiding. Six analytes were targeted for SCFA analysis, namely, acetic acid (Dr. Ehrenstorfer, Germany), propionic acid (Dr. Ehrenstorfer, Germany), butyric acid (Dr. Ehrenstorfer, Germany), isobutyric acid (Supelco, United States), valeric acid (Nu-Chek, United States), and isovaleric acid (Sigma-Aldrich, United States). Feces were homogenized in 1.0 mL of ultrapure water containing an internal standard, 2,2-dimethylbutyric acid (Dr. Ehrenstorfer, Germany). After centrifugation, the supernatant was transferred into a new tube. Then, 10 μL of 50% sulfuric acid and 0.5 g of sodium sulfate (Macklin, China) were added to the tube along with analytically pure diethyl ether (2 mL). The solution was vortexed for 1 min and then centrifuged for 10 min at room temperature. The ether layer was collected for gas chromatography with mass selective detection (5977B GC/MSD, Agilent Technologies, Santa Clara, CA, United States) measurement (Supplementary Table S2). The GC/MS data were acquired and analyzed using MassHunter Workstation software (Agilent Technologies) running on Windows 7 (Microsoft, Redmond, WA, United States). The concentrations of fecal SCFAs were calculated with the use of external standards and are expressed as micromoles per gram of wet feces.

### Intestinal Permeability Biomarker Quantification by ELISA

Intestinal permeability was determined as the serum levels of D-lactate (D-LA), intestinal fatty acid-binding protein (iFABP), lipopolysaccharide (LPS), and lipopolysaccharide-binding protein (LBP), as reported before (22, 23).

After centrifuging the blood samples, plasma-EDTA was stored at −80°C until measurement. Plasma samples used for D-LA, iFABP, LPS and LBP quantification were analyzed in duplicate using ELISA kits (Bio-swamp Life Science, Wuhan, Hubei, China) following the manufacturer’s protocols.

### Statistical Analysis

The continuous non-parametric data are presented as medians (interquartile ranges, IQRs) and were analyzed using Mann-Whitney U or Wilcoxon tests. The continuous parametric data are presented as the means (standard deviations, SDs) and were analyzed with Student’s *t* tests. The categorical data are presented as numbers (percentages, %) and were analyzed using chi-squared tests. For microbial analysis, QIIME analysis was additionally performed using the Adonis test as previously described (12). Correlations between variables were determined with Spearman’s rank correlation test. SPSS version 20 (Statistical Package for Social Sciences, Chicago, IL, United States) was used for statistical analysis. Two-tailed *p* values of<0.05 were considered statistically significant. The figures were generated using GraphPad Prism 7 or R version 3.4.3^2^.

## Results

### Prevalence of Pathogens in Patients With Encephalitis

**FIGURE 1:**
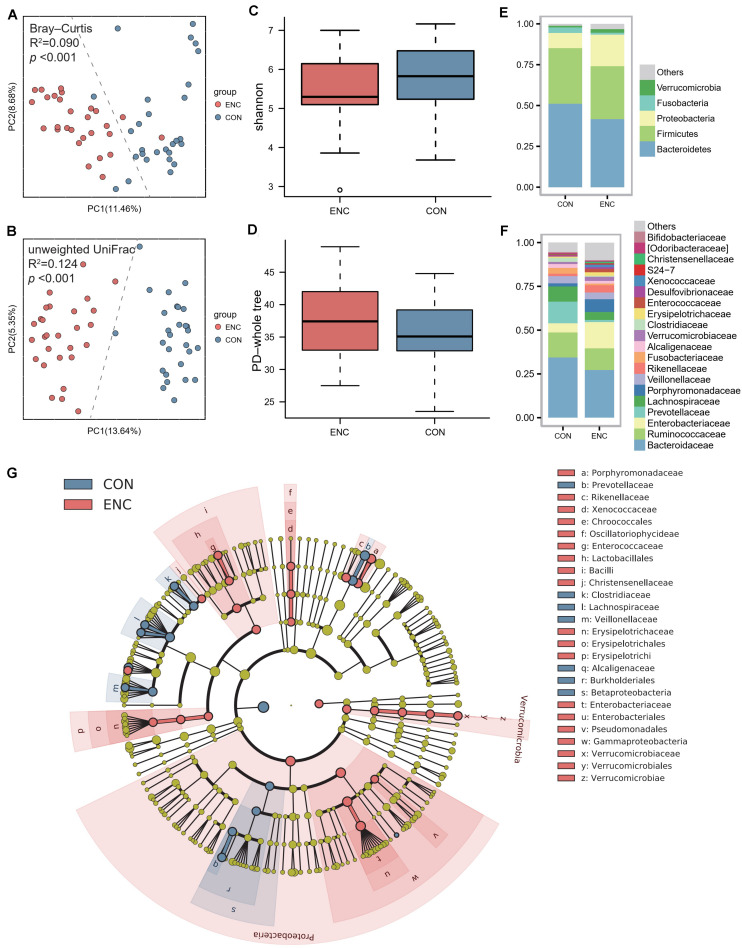
The gut microbiota composition of encephalitis patients was significantly different from that of healthy subjects. **(A,B)** The β-diversity in the ENC and CON groups was calculated by the Bray-Curtis distance **(A)** and unweighted UniFrac distance **(B)** and is shown in the PCoA plot (Adonis test, Bray-Curtis distance, *R*^2^ = 0.090, *p* < 0.001; unweighted UniFrac distance, *R*^2^ = 0.124, *p* < 0.001). Each point represents the composition of the intestinal microbiota of one participant. **(C,D)** The α-diversity of the microbiota, presented as the Shannon index **(C)** and PD-whole tree index **(D)**, was calculated from samples from encephalitis patients and healthy subjects (Mann-Whitney *U* test, Shannon index, *p* = 0.098; PD-whole tree index, *p* = 0.350). The boxplots display the 95% CIs, and the points lying outside the whiskers are referred to as outliers. **(E,F)** Average relative abundances of the predominant bacterial taxa at the phylum **(E)** and family **(F)** levels in the ENC and CON groups. **(G)** Cladogram based on LEfSe results of the CON and ENC groups. The red points represent the increased taxa in ENC group, while the blue points represent the increased taxa in CON group. ENC, patients with encephalitis; CON, healthy subjects serving as controls.

Fecal samples were collected from 28 encephalitis (ENC) patients (median age 46 years; 82.1% male; median GCS 6.5; median SOFA 6.5; median APACHE-II 14.5; median IOS 12.5). The clinical information of all encephalitis patients is shown in Table 1. Until 180 days follow-up, there were 11 patients were alive, whereas 17 patients were deceased. Twenty-eight healthy subjects served as the healthy controls (CON) and had fecal samples collected once. A principal coordinate analysis (PCoA) plot showed a significant difference in β-diversity [Bray-Curtis distance (Figure 1A) and unweighted UniFrac distance (Figure 1B)] between the ENC and CON groups (Adonis test, *p* > 0.05). The α-diversity, including Shannon, Chao1, PD-whole tree, observed species, and Simpson indexes, did not show significant differences between these two groups (Mann-Whitney *U* test, *p* > 0.05) (Figures 1C,D and Supplementary Table S3). As indicated by taxonomic summary (Figures 1E,F) and cladogram based on LEfSe analysis (Figure 1G), the relative abundances of the phyla Proteobacteria, Deferribacteres and Verrucomicrobia were higher in the neuroICU group than in the HC group. At the family level, Enterobacteriaceae, Porphyromonadaceae, Enterococcaceae, Verrucomicrobiaceae, Rikenellaceae and Lactobacillaceae were enriched in the neuroICU group.

**TABLE 1:** Clinical features obtained from 28 encephalitis patients.

Patient	GCS	SOFA	APACHE-II	Ventilator Support	IOS	180d Outcome
ENC01	6	15	18	Yes	66	Dead
ENC02	4	10	28	Yes	5	Dead
ENC03	3	11	20	Yes	99	Dead
ENC04	5	10	18	Yes	68	Dead
ENC05	6	8	18	Yes	37	Dead
ENC06	5	5	26	No	45	Survived
ENC07	7	9	12	No	22	Dead
ENC08	7	4	15	No	13	Dead
ENC09	6	9	22	Yes	20	Dead
ENC10	7	4	12	No	11	Survived
ENC11	8	10	15	Yes	22	Survived
ENC12	4	5	21	No	66	Survived
ENC13	10	2	24	No	16	Dead
ENC14	5	6	18	No	4	Survived
ENC15	9	5	9	No	5	Survived
ENC16	5	7	15	Yes	16	Dead
ENC17	8	7	11	Yes	30	Survived
ENC18	9	3	12	No	8	Survived
ENC19	8	6	18	Yes	16	Dead
ENC20	3	11	19	Yes	21	Dead
ENC21	9	10	20	Yes	17	Dead
ENC22	5	9	13	No	6	Survived
ENC23	5	8	20	Yes	26	Survived
ENC24	10	7	20	No	3	Dead
ENC25	8	3	10	No	17	Survived
ENC26	8	12	19	Yes	7	Dead
ENC27	7	6	14	No	5	Dead
ENC28	6	10	21	Yes	8	Dead

*GCS, Glasgow Coma Scale; APACHE-II, Acute Physiology and Chronic Health Evaluation-II; SOFA, Sequential Organ Failure Assessment; IOS, length of stay in intensive care units; M, male; F, female.*

To evaluate differences in microbial composition in the feces obtained from patients and controls, we compared the relative abundances in both groups, represented by read percentages (Table 2). The significantly enriched taxa in the patient group were the phylum *Proteobacteria*, class *Bacilli*, class *Gammaproteobacteria*, order *Lactobacillales*, order *Erysipelotrichales*, order *Enterobacteriales*, family *Porphyromonadaceae*, family *Enterobacteriaceae*, genus *Parabacteroides*, and genus *Oscillospira*. The significantly depleted taxa in the patient group were the class *Betaproteobacteria*, order *Burkholderiales*, family *Lachnospiraceae*, genus *Prevotella*, genus *Faecalibacterium*, genus *Ruminococcus*, and genus *Sutterella*.

**TABLE 2:** Significantly discriminative taxa between the twenty-eight Encephalitis patients and healthy subjects determined by Mann-Whitney *U* tests.

Taxa	Encephalitis, M (IQR)	Control, M (IQR)	*p* value
**The taxa increased in encephalitis patients**			
Phylum *Proteobacteria*	0.138 (0.084–0.241)	0.068 (0.052–0.116)	0.001
Class *Bacilli*	0.015 (0.006–0.028)	0.004 (0.002–0.007)	<0.001
Class *Gammaproteobacteria*	0.096 (0.046–0.195)	0.026 (0.016–0.074)	<0.001
Order *Lactobacillales*	0.014 (0.006–0.027)	0.003 (0.002–0.007)	<0.001
Order *Erysipelotrichales*	0.008 (0.004–0.034)	0.004 (0.003–0.006)	0.011
Order *Enterobacteriales*	0.081 (0.041–0.191)	0.024 (0.013–0.058)	<0.001
Family *Porphyromonadaceae*	0.042 (0.026–0.110)	0.016 (0.010–0.022)	<0.001
Family *Enterobacteriaceae*	0.081 (0.041–0.191)	0.024 (0.013–0.058)	<0.001
Genus *Parabacteroides*	0.041 (0.026–0.110)	0.016 (0.010–0.021)	<0.001
Family Rikenellaceae, genus *undefined*	0.024 (0.009–0.043)	0.010 (0.004–0.017)	0.001
Family S24-7, genus *undefined*	0.007 (0.005–0.009)	0.002 (0.001–0.005)	0.001
Genus *Oscillospira*	0.011 (0.008–0.015)	0.006 (0.004–0.008)	0.003
Family *Enterobacteriaceae*, genus *undefined*	0.076 (0.034–0.189)	0.022 (0.012–0.056)	<0.001
Genus *Akkermansia*	0.006 (0.003–0.016)	0.003 (0.001–0.012)	0.063
**The taxa decreased in encephalitis patients**			
Phylum *Bacteroidetes*	0.442 (0.221–0.541)	0.510 (0.388–0.644)	0.078
Class *Clostridia*	0.236 (0.145–0.372)	0.315 (0.221–0.452)	0.075
Class *Betaproteobacteria*	0.012 (0.007–0.017)	0.022 (0.013–0.031)	0.011
Order *Clostridiales*	0.236 (0.145–0.372)	0.315 (0.221–0.452)	0.075
Order *Burkholderiales*	0.012 (0.007–0.017)	0.022 (0.013–0.031)	0.011
Family *Lachnospiraceae*	0.037 (0.022–0.059)	0.068 (0.056–0.111)	<0.001
Genus *Prevotella*	0.007 (0.003–0.014)	0.025 (0.016–0.106)	<0.001
Family *Lachnospiraceae*, genus *undefined*	0.022 (0.013–0.035)	0.035 (0.023–0.045)	0.010
Genus *Faecalibacterium*	0.008 (0.004–0.028)	0.040 (0.022–0.069)	0.001
Genus *Ruminococcus*	0.004 (0.002–0.009)	0.009 (0.004–0.016)	0.030
Genus *Sutterella*	0.011 (0.006–0.016)	0.021 (0.012–0.030)	0.005

*M, median; IQR, interquartile range.*

The PICRUSt algorithm was performed to identify which pathway or mechanism is affected, based on microbial change (Supplementary Figure S1). As shown in the results, the pathways upregulated in ENC group including Transport and Catabolism, Immune System Diseases, Folding, Sorting and Degradation, Energy Metabolism, Cancers, Lipid Metabolism, Amino Acid Metabolism, Metabolism of Terpenoids and Polyketides, Enzyme Families, Genetic Information Processing, Signaling Molecules and Interaction, Metabolic Diseases, Excretory System, Transcription, Metabolism, Cellular Processes and Signaling, Metabolism of Other Amino Acids, Carbohydrate Metabolism, Poorly Characterized, Membrane Transport, Neurodegenerative Diseases, Xenobiotics Biodegradation and Metabolism, Signal Transduction, Infectious Diseases.

### The Correlations Between Microbial Indexes and Clinical Parameters

To identify correlations between fecal microbiota composition and health status, we first examined the correlations among microbial α-diversity indexes (Shannon, PD-whole tree, Chao1, observe species, Simpson) and clinical data (Supplementary Figure S2). The blood-brain barrier permeability is presented as the CSAR, which can reflect the degree of cerebral inflammation. PD-whole tree was positively correlated with the serum concentrations of potassium (*r* = 0.391, *p* = 0.040) and S100β (*r* = 0.394, *p* = 0.038) but negatively correlated with levels of total bilirubin (*r* = −0.386, *p* = 0.042). Observed species was significantly correlated with S100β levels (*r* = 0.433, *p* = 0.021). The Shannon, PD-whole tree and observed species indexes had positive correlations with the CSAR (*r* = 0.468, *p* = 0.018; *r* = 0.449, *p* = 0.024; and *r* = 0.395, *p* < 0.05, respectively).

**FIGURE 2:**
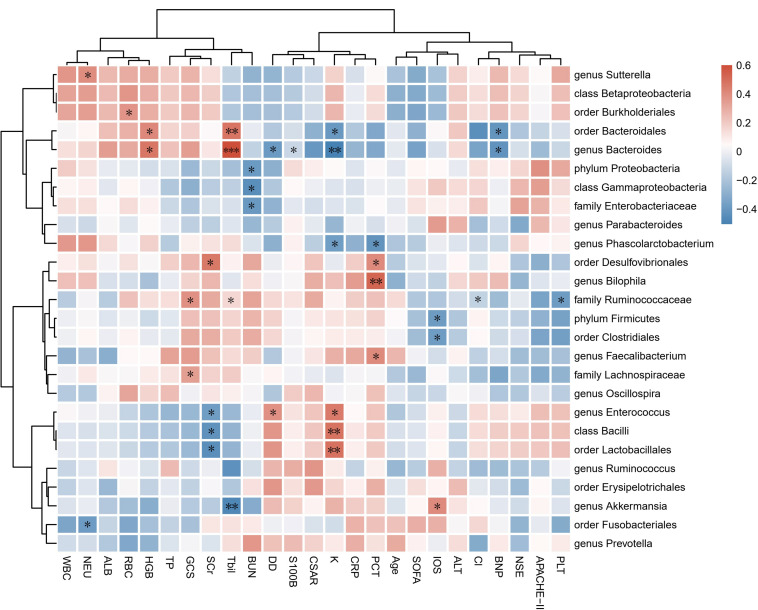
Associations of gut microbial taxa with clinical indexes. Heat map of Spearman’s rank correlation coefficient among 17 clinical indexes and 26 taxa with abundances higher than 0.1%. *n* = 28; ^∗^*p* < 0.05; ^∗∗^*p* < 0.01; ^∗∗∗^*p* < 0.001; Spearman’s rank correlation. WBC, white blood cell count; NEU, neutrophil count; RBC, red blood cell count; HGB, hemoglobin; PLT, platelet count; ALT, alanine aminotransferase; Tbil, total bilirubin; TP, total protein; ALB, albumin; Cl, serum chlorine, K, serum potassium; BUN, blood urea nitrogen; SCr, serum creatinine; CRP, C-reactive protein; PCT, procalcitonin; DD, D-dimer; BNP, brain natriuretic peptide; NSE, neuron-specific enolase; S100B, S100 calcium-binding protein B; CSAR, cerebrospinal fluid albumin-to-serum albumin ratio; GCS, Glasgow Coma Scale; SOFA, Sequential Organ Failure Assessment; APACHE-II, Acute Physiology and Chronic Health Evaluation-II; IOS, length of stay in the ICU.

Correlation analysis was subsequently performed among clinical parameters and relative abundances of bacterial groups detected in the feces of encephalitis patients (Figure 2). A positive correlation of the family *Ruminococcaceae* reads with GCS score was observed (*r* = 0.384, *p* = 0.044). The phylum *Firmicutes* and order *Clostridiales* were positively associated with IOS (*r* = −0.387, *p* = 0.042 and *r* = −0.383, *p* = 0.044, respectively). The genus *Akkermansia* showed negative correlations with IOS (*r* = 0.404, *p* = 0.033).

**FIGURE 3:**
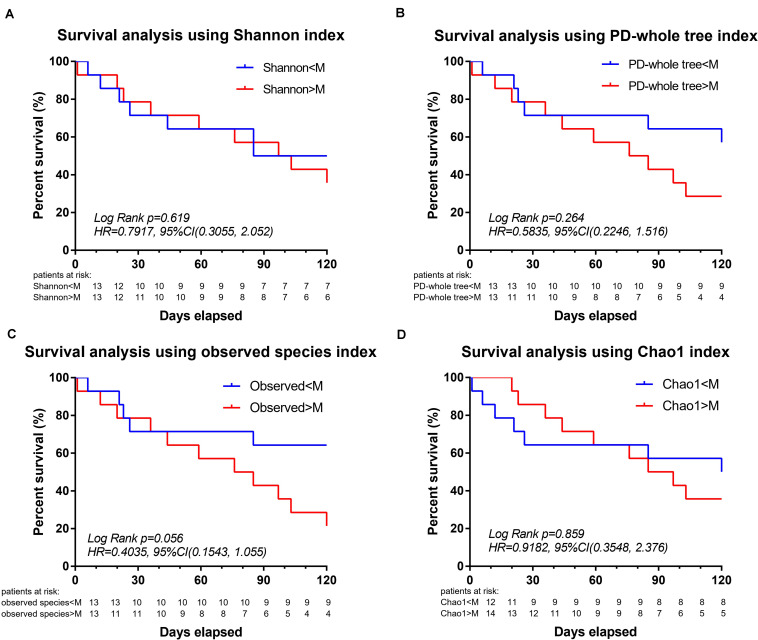
Decreased intestinal microbiota diversity in encephalitis patients is not associated with survival in an exploratory setting. Based on the α-diversities in healthy subjects, the patient cohort was split into two groups: α-diversity < Median and α-diversity > Median, for which a 120-day Kaplan–Meier survival plot is shown. Twenty-eight encephalitis patients were divided based on the median of Shannon index [**A**, Log Rank *p* = 0.619, HR = 0.7917, 95%CI = (0.3055, 2.052)], PD-whole tree index [**B**, Log Rank *p* = 0.264, HR = 0.5835, 95%CI = (0.2246, 1.516)], observed species [**C**, Log Rank *p* = 0.056, HR = 0.4035, 95%CI = (0.1543, 1.055)], Chao1 index (**D**, Log Rank *p* = 0.859, HR = 0.9182, 95%CI = (0.3548, 2.376)], successively. M, median. Numbers below the curve were patients at risk per group.

Survival analysis was further performed to explore the association between mortality and microbial diversity (Figure 3). When the cohort was divided into two groups with low bacterial diversity (α-diversity < Median, *n* = 14) and high diversity (α-diversity > Median, *n* = 14), there was no intergroup difference in the short-term mortality. However, when the patients were divided into two groups based on the median of observed species, the survival analysis had a trend toward significance [Log Rank *p* = 0.056, HR = 0.4035, 95%CI = (0.1543, 1.055)].

### Fecal Short-Chain Fatty Acid Levels Are Decreased in Encephalitis Patients

**FIGURE 4:**
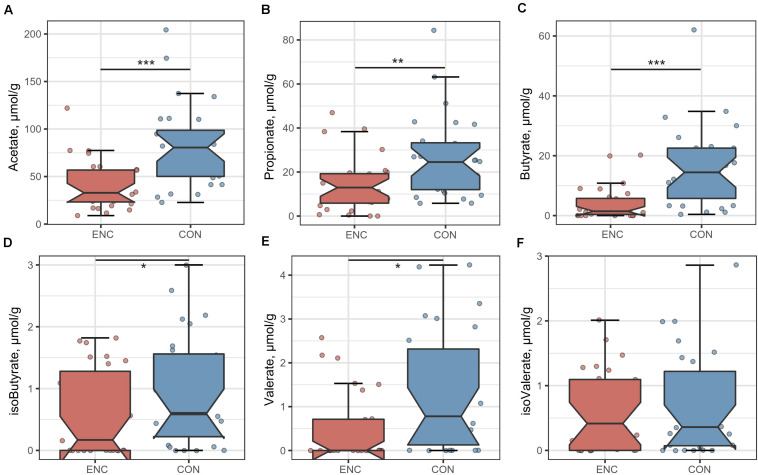
Comparison of six fecal SCFA levels between encephalitis patients and healthy controls. **(A)** acetate; **(B)** propionate; **(C)** butyrate; **(D)** isobutyrate; **(E)** valerate; **(F)** isovalerate. *p < 0.05; **p < 0.01; ***p < 0.001; Mann-Whitney *U* test. ENC, patients with encephalitis; CON, healthy subjects serving as controls.

To evaluate the SCFAs in fecal samples from encephalitis patients and healthy subjects, we quantified the fecal concentrations of acetate, propionate, butyrate, isobutyrate, valerate and isovalerate by GC-MS (Figure 4). The concentrations of acetate, propionate and butyrate were significantly increased in the fecal samples from encephalitis patients (acetate: 41.11 ± 25.71 μmol/g; propionate: 14.44 ± 12.28 μmol/g; butyrate: 4.144 ± 5.509 μmol/g) compared with those in samples from healthy subjects (acetate: 82.64 ± 43.01 μmol/g; propionate: 26.48 ± 18.34 μmol/g; butyrate: 15.84 ± 13.41 μmol/g). Isobutyrate, valerate and isovalerate were nearly undetectable in the vast majority of patient and control samples.

Spearman’s tests were performed to identify correlations between fecal SCFAs and clinical parameters. Results were shown in Supplementary Figure S3. We found that acetate was negatively correlated with age (*r* = −0.433, *p* = 0.027), BUN (*r* = −0.498, *p* = 0.010) and CRP (*r* = −0.432, *p* = 0.028); propionate showed negative correlation with age (*r* = −0.532, *p* = 0.005); butyrate was negatively correlated with CRP (*r* = −0.433, *p* = 0.027), age (*r* = −0.534, *p* = 0.005), CSAR (*r* = −0.539, *p* = 0.008) and D-LA (*r* = −0.390, *p* = 0.049) while positively correlated with ALB (*r* = 0.488, *p* = 0.011).

### Gut Permeability Was Increased in Encephalitis Patients

**FIGURE 5:**
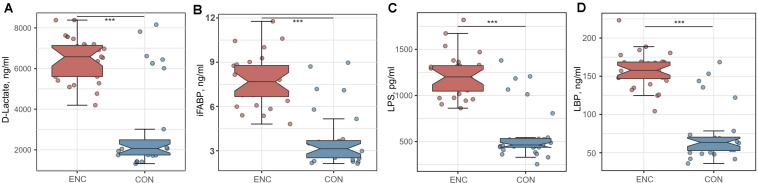
Comparison of intestinal integrity biomarkers between encephalitis patients and healthy controls. **(A)** D-lactate; **(B)** iFABP; **(C)** LPS; **(D)** LBP. ***p < 0.001; Mann-Whitney *U* test. iFABP, intestinal fatty acid-binding protein; LPS, lipopolysaccharide; LBP, lipopolysaccharide-binding protein; ENC, patients with encephalitis; CON, healthy subjects serving as controls.

To evaluate intestinal permeability in encephalitis patients and healthy controls, we quantified the plasma concentrations of D-LA, iFABP, LPS and LBP, which were previously reported as intestinal integrity biomarkers (22–24). The concentrations of D-LA, iFABP, LPS, and LBP were significantly higher in plasma samples from encephalitis patients (D-LA: 6430.2 ± 1056.2 ng/mL; iFABP: 7.779 ± 1.714 ng/mL; LPS: 1218.3 ± 229.9 pg/mL; LBP: 157.9 ± 23.3 ng/mL) than in samples from healthy subjects (D-LA: 3006.6 ± 2123.4 ng/mL; iFABP: 3.813 ± 1.952 ng/mL; LPS: 585.7 ± 297.4 pg/mL; LBP: 73.5 ± 35.8 ng/mL), indicating that intestinal mucosal integrity was significantly reduced during cerebral inflammation (Figure 5).

Spearman’s correlation analyses were further performed to identify correlations among intestinal integrity biomarkers and clinical parameters. Results were shown in Supplementary Figure S4. The iFABP showed positive correlations with LPS (*r* = 0.586, *p* = 0.001) and S100B (*r* = 0.439, *p* = 0.019); LPS correlated positively with CSAR (*r* = 0.435, *p* = 0.030); LBP has negative correlation with TP (*r* = −0.411, *p* = 0.030).

## Discussion

In this observational pilot study, the microbiome of many encephalitis patients differed substantially from that of a healthy population, and the disruption of the microbial community may have resulted in the dysbiosis of SCFAs. We documented increases in the abundances of the phylum *Proteobacteria* as well as other pathogens present relative to those in healthy adults. Fecal acetate, propionate and butyrate concentrations in patients with encephalitis decreased significantly in comparison with those in the healthy volunteers. In addition, increased levels of gut microbial components or products were detected in the systemic circulation, indicating that the dysbiosis of the commensal flora and lack of SCFAs may have been responsible for the intestinal mucosal injury and gut permeability elevation. A set of clinical parameters, especially the CSAR representing the blood-brain barrier, were associated with microbiome indexes or specific taxon abundances. This study provides the first *in vivo* evidence that an altered gut flora and the concentrations of SCFAs are associated with worse health status. The results of these explorations suggest that larger prospective studies should be undertaken to monitor the microbiome of patients with inflammatory disease. Furthermore, new therapeutic interventions (e.g., bacteriophage therapy) targeting gut bacteria and protecting gut function may be a potential option to improve the outcome of these patients.

Pivotal to many biological functions in the human body is the composition of the healthy microbiota, which affects various physiological processes, including the development of the digestive tract (25), gut barrier function and integrity (26), the immune response (27), and the homeostasis of the CNS. The effects of the gut microbiota on the brain include regulating neurotransmitters, neurotrophic factors and synaptogenesis, as well as maintaining BBB integrity (28, 29). Our study used culture-independent techniques to confirm and characterize the significant dysbiosis in encephalitis, as illustrated by a PCoA plot. Although we did not detect a significant difference in α-diversities between patients and healthy groups, this result is likely underpowered owing to the sophisticated calculations of microbial diversity indexes and the relatively small number of patients enrolled. We observed enrichment of disease-promoting pathogens, such as the family Enterobacteriaceae (30), in encephalitis patients. Conversely, some taxa that were depleted in the patient group, such as the genus *Faecalibacterium*, were previously believed to confer antiinflammatory benefits (31). These findings likely reflect numerous variables, including derangements in host physiology, multiple treatment exposures, and the presence of nosocomial pathogens. Moreover, pathogens can inhibit the growth of other bacteria, a phenomenon referred to as “colonization resistance” (32). Unexpectedly, some probiotics [the genera *Parabacteroides* (33) and *Akkermansia* (34)] were found to be enriched in the patient group, whereas several pathogens [the genus *Prevotella* (35)] were depleted. This result can likely be attributed to the controversial role of taxa. As the 16S rRNA sequence cannot definitively assign identity at the species or strain level, further exploration of the microbiome will require targeted sequencing methods, ideally with functional metagenomics.

To investigate the possible link between bacterial indexes and illness status, we explored the association among clinical parameters and both microbiome indexes and specific taxa. The phyla *Proteobacteria* and *Firmicutes* were related to disease severity, as reflected by APACHE-II and IOS, respectively. Bacterial α-diversity indexes, including PD-whole tree, Shannon index and observed species, were associated with some clinical parameters, especially the CSAR. The CSAR is one of the most informative parameters for BBB integrity in cases of CNS disease (36). The BBB acts as a gatekeeper to control the passage and exchange of molecules and nutrients between the circulatory system and the brain parenchyma. Persistent vulnerability of an impaired BBB caused by inflammation (37) would compromise the CNS. Currently, no effective drugs are available for direct treatment of BBB dysfunction. Repairing BBB function by the gut flora is a potential therapeutic target for the development of new-generation antiencephalitis drugs.

In our study, the fecal concentrations of acetate, propionate and butyrate in the patients with encephalitis were significantly lower than those in healthy subjects. Derived from intestinal microbial fermentation of dietary fiber, SCFAs are the main energy source of colonocytes, making them crucial to gastrointestinal health (38). As reported before, SCFA formation is regulated mainly by substrate availability and bacterial species composition (39). First, in terms of the microbiota, beneficial bacteria counts in the patients with encephalitis were significantly lower than those in the healthy volunteers. Although the identification of butyrate-producing microorganisms is still under investigation (40), some known organic acid-producing bacteria, including the family *Lachnospiraceae* (41), genus *Ruminococcus* (42), and genus *Faecalibacterium* (43), were depleted in encephalitis patients, as quantified by 16S rRNA sequencing. Second, it is possible that fermentation substrates, such as soluble dietary fiber (44), may have been relatively reduced in encephalitis patients, contributing to the low SCFA levels. These two hypotheses behind the decrease in SCFA levels in encephalitis patients should be further investigated in future research. SCFAs are taken up directly into the bloodstream and transported to various organs, including the brain (45), where they modulate tissue development and function (46). As an inhibitor of histone deacetylases (HDACs), butyrate exhibits antiinflammatory and neuroprotective effects through multiple mechanisms, including enhancing neurogenesis and reducing proinflammatory cytokine levels (47–51). Recent studies have shown that various G protein-coupled receptors (GPRs) mediate SCFA activities and affect the inflammatory response. SCFAs activate GPR41 and GPR43 on intestinal epithelial cells, leading to mitogen-activated protein kinase signaling and the production of chemokines and cytokines (52). Moreover, butyrate promotes antiinflammatory properties via the GPR109a signaling pathway (53, 54). These results suggest that the decrease in SCFA levels observed in the present study could be conducive to sustained inflammation in encephalitis patients by mechanisms related to HDACs and GPRs.

Short-chain fatty acids, especially butyrate, are an energy source for colon epithelial cells and have been shown to regulate intestinal motility (55, 56). Physiological concentrations of SCFAs regulate intestinal barrier function by decreasing paracellular permeability and increasing transepithelial electrical resistance (57). Butyrate was demonstrated to improve gut barrier function by stimulating the production of mucin, antimicrobial peptides, and tight junction proteins (58). Regulation of occludin expression by the intestinal microbiota has been reported in the intestinal epithelial barrier (59) and blood-testis barrier (60). Clostridial clusters make a great contribution to gut homeostasis by preserving gut barrier functions and exerting immunomodulatory and antiinflammatory properties (61). It was speculated that alteration of the gut flora and SCFA levels shapes the leaky gut, which subsequently results in the translocation of microbial components, such as LPS, into systemic circulation, activating the inflammatory response or increasing BBB permeability. The restoration of healthy microbes or SCFAs can potentially be a future treatment.

As a result of incomplete understanding of the pathological mechanisms combined with individual variations in the immune response to causative agents, treatment of encephalitis remains a great challenge for physicians. This pilot study seeks to explain the changes in the intestine in encephalitis patients and highlights the possible association between the gut and brain. Previous studies have shown that maintenance of commensal “healthy microbes” or modulation of SCFAs may exert beneficial effects via multiple pathways, including modulation of immune cell proliferation, suppression of pathogenic microbes by antimicrobial factors, and gut epithelial barrier protective effects (62–64). Administration of SCFAs (65) or prebiotics (66) has been reported as an effective therapy to increase intestinal SCFA levels. The clinical effect of increasing SCFA levels by synbiotic administration has also been demonstrated (67). In previous research, patients with sepsis benefited from synbiotic treatment, having a significantly lower incidence of infectious complications than those without synbiotic consumption (68). This evidence, together with this study, suggests that patients may benefit from intestinal therapeutics focused on improvement of the gut microbiome and SCFA levels. As the gut is hypothesized to play a central role in the progression of severe inflammation (69), creative new approaches to repopulate the normal “health-promoting” microbiome may present opportunities to improve outcomes in these encephalitis patients.

As an observational pilot study, this study has several limitations. First, this study did not aim to reveal the precise signaling mechanisms through which gut microbiota interacts with encephalitis but provided a first glimpse into the superficial layer of gut-brain communication. In addition, the consequences of altered flora on brain function throughout the pathophysiological process of encephalitis are still unknown. Therefore, the results should be interpreted cautiously until additional advanced data are acquired to clarify the underlying mechanisms. The next target for our subsequent study is trying to maintain the commensal flora in a mouse model and, in this way, attain any associated clinical benefits. This approach may hopefully explain a causal relationship in the gut-brain axis. Second, the number of fecal samples as well as enrolled patients remains relatively modest. Due to the small sample size, we evaluated the integrated data of patients with various etiologies of encephalitis, limiting the insights gained from analyses. Similarly, the correlation analyses between microbiota indexes and clinical parameters were not controlled for multiple confounders and, as such, merit replication in larger cohorts. Third, the single fecal sample from each patient studied here could not provide a dynamic view of the microbiota. A few patients could be sampled twice because some died or were transferred to other ward for better treatment. We believe that the gut flora and SCFAs may change along with the recovery or deterioration of the disease. Longitudinal analyses should be considered as a subject of our future studies. Finally, the microbiome and SCFAs in the cecal matter differ from those detected in fecal samples (70). However, it is not possible to obtain cecal samples from the human body; therefore, stool is used. The combination of these limitations makes it challenging to establish a rigorous statistical analysis in this study.

Taken together, our data demonstrate that disruption of the gut microbiota was observed in encephalitis patients, which manifested as pathogen dominance and health-promoting commensal microbe depletion. This study adds to the emerging literature describing dysbiosis in inflammatory diseases of the CNS. We also identified reduced intestinal barrier integrity, probably as a result of the dysbiosis of the gut microbiota and SCFAs. The disease severity and the degree of brain damage may have associations with the gut microbiota or its metabolites. Numerous questions remain to be answered, including the following. How does the gut microbiota affect the blood-brain barrier? What is the mechanism by which an increase in pathogen abundance could affect the inflammatory system? Further studies, such as fecal microbiota transplantation experiments, are needed to confirm the results in this study and to evaluate the causal relationship in the gut-brain axis.

## Data Availability Statement

The data has been uploaded to the European Nucleotide Archive – PRJEB39342. Other raw data supporting the conclusions of this article will be made available by the authors, without undue reservation, to any qualified researcher.

## Ethics Statement

The studies involving human participants were reviewed and approved by the Medical Ethics Committee of Nanfang Hospital. The patients/participants provided their written informed consent to participate in this study.

## Author Contributions

RX participated in patients enrollment and sample collection. CT participated in fecal microbe DNA extraction and V3/V4 amplification. YH provided the support for Illumina platform sequencing and manuscript writing and revision. QW participated in library construction and sequencing. HW participated in short-chain fatty acids determination and manuscript writing. JY provided the support for experimental conception, control samples collection, patients enrollment, and manuscript revision. All authors contributed to the article and approved the submitted version.

## Conflict of Interest

The authors declare that the research was conducted in the absence of any commercial or financial relationships that could be construed as a potential conflict of interest.

## Footnotes

1 http://huttenhower.sph.harvard.edu/galaxy

2 https://www.r-project.org/

## References

[1] Granerod J Crowcroft NS. The epidemiology of acute encephalitis. *Neuropsychol Rehabil.* (2007) 17:406–28. 10.1080/09602010600989620 17676528

[2] Granerod J Ambrose HE Davies NW Clewley JP Walsh AL Morgan D Causes of encephalitis and differences in their clinical presentations in England: a multicentre, population-based prospective study. *Lancet Infect Dis.* (2010) 10:835–44. 10.1016/S1473-3099(10)70222-X 20952256

[3] Goodfellow JA Mackay GA. Autoimmune encephalitis. *J R Coll Physicians Edinb.* (2019) 49:287–94. 10.4997/JRCPE.2019.407 31808454

[4] Toledano M Davies NWS. Infectious encephalitis: mimics and chameleons. *Pract Neurol.* (2019) 19:225–37. 10.1136/practneurol-2018-002114 30878971

[5] Ellul M Solomon T. Acute encephalitis–diagnosis and management. *Clin Med (Lond).* (2018) 18:155–9. 10.7861/clinmedicine.18-2-155 29626021 PMC6303463

[6] Mailles A Stahl JP Bloch KC. Update and new insights in encephalitis. *Clin Microbiol Infect.* (2017) 23:607–13. 10.1016/j.cmi.2017.05.002 28501667

[7] Beaman MH. Community-acquired acute meningitis and encephalitis: a narrative review. *Med J Aust.* (2018) 209:449–54. 10.5694/mja17.01073 30309300

[8] Disease GBD Injury I Prevalence C. Global, regional, and national incidence, prevalence, and years lived with disability for 310 diseases and injuries, 1990-2015: a systematic analysis for the Global Burden of Disease Study 2015. *Lancet.* (2016) 388:1545–602. 10.1016/S0140-6736(16)31678-6 27733282 PMC5055577

[9] Mortality GBD, Causes of Death Collaborators. Global, regional, and national life expectancy, all-cause mortality, and cause-specific mortality for 249 causes of death, 1980-2015: a systematic analysis for the Global Burden of Disease Study 2015. *Lancet.* (2016) 388:1459–544. 10.1016/S0140-6736(16)31012-1 27733281 PMC5388903

[10] Mcdonald D Ackermann G Khailova L Baird C Heyland D Kozar R Extreme dysbiosis of the microbiome in critical illness. *mSphere.* (2016) 1:e00199-16. 10.1128/mSphere.00199-16 27602409 PMC5007431

[11] Xia GH You C Gao XX Zeng XL Zhu JJ Xu KY Stroke dysbiosis index (SDI) in gut microbiome are associated with brain injury and prognosis of stroke. *Front Neurol.* (2019) 10:397. 10.3389/fneur.2019.00397 31068891 PMC6491752

[12] Yin J Liao SX He Y Wang S Xia GH Liu FT Dysbiosis of Gut microbiota with reduced trimethylamine-N-oxide level in patients with large-artery atherosclerotic stroke or transient ischemic attack. *J Am Heart Associat.* (2015) 4:e002699. 10.1161/JAHA.115.002699 26597155 PMC4845212

[13] Hindson J. Multiple sclerosis: a possible link between multiple sclerosis and gut microbiota. *Nat Rev Neurol.* (2017) 13:705. 10.1038/nrneurol.2017.142 28960186

[14] Schepici G Silvestro S Bramanti P Mazzon E. The gut microbiota in multiple sclerosis: an overview of clinical trials. *Cell Transplant.* (2019) 28:1507–27. 10.1177/0963689719873890 31512505 PMC6923550

[15] Gong J Qiu W Zeng Q Liu X Sun X Li H Lack of short-chain fatty acids and overgrowth of opportunistic pathogens define dysbiosis of neuromyelitis optica spectrum disorders: a Chinese pilot study. *Mult Scler.* (2018) 25:1316–25. 10.1177/1352458518790396 30113252

[16] Di Stefano A Alcantarini C Atzori C Lipani F Imperiale D Burdino E Cerebrospinal fluid biomarkers in patients with central nervous system infections: a retrospective study. *CNS Spectr.* (2019) 25:402–8. 10.1017/S1092852919000981 31130152

[17] Peng X Yu KQ Deng GH Jiang YX Wang Y Zhang GX Comparison of direct boiling method with commercial kits for extracting fecal microbiome DNA by Illumina sequencing of 16S rRNA tags. *J Microbiol Methods.* (2013) 95:455–62. 10.1016/j.mimet.2013.07.015 23899773

[18] Caporaso JG Kuczynski J Stombaugh J Bittinger K Bushman FD Costello EK QIIME allows analysis of high-throughput community sequencing data. *Nature Methods.* (2010) 7:335–6. 10.1038/nmeth.f.303 20383131 PMC3156573

[19] Lozupone C Hamady M Knight R. UniFrac–an online tool for comparing microbial community diversity in a phylogenetic context. *BMC Bioinformatics.* (2006) 7:371. 10.1186/1471-2105-7-371 16893466 PMC1564154

[20] Lozupone C Knight R. UniFrac: a new phylogenetic method for comparing microbial communities. *Appl Environ Microbiol.* (2005) 71:8228–35. 10.1128/AEM.71.12.8228-8235.2005 16332807 PMC1317376

[21] Segata N Izard J Waldron L Gevers D Miropolsky L Garrett WS Metagenomic biomarker discovery and explanation. *Genome Biol.* (2011) 12:R60. 10.1186/gb-2011-12-6-r60 21702898 PMC3218848

[22] Grootjans J Thuijls G Verdam F Derikx JP Lenaerts K Buurman WA. Non-invasive assessment of barrier integrity and function of the human gut. *World J Gastrointest Surg.* (2010) 2:61–9. 10.4240/wjgs.v2.i3.61 21160852 PMC2999221

[23] Camara-Lemarroy CR Silva C Greenfield J Liu WQ Metz LM Yong VW. Biomarkers of intestinal barrier function in multiple sclerosis are associated with disease activity. *Mult Scler.* (2019) 18:1352458519863133. 10.1177/1352458519863133 31317818

[24] Zhou X Li J Guo J Geng B Ji W Zhao Q Gut-dependent microbial translocation induces inflammation and cardiovascular events after ST-elevation myocardial infarction. *Microbiome.* (2018) 6:66. 10.1186/s40168-018-0441-4 29615110 PMC5883284

[25] Hooper LV. Bacterial contributions to mammalian gut development. *Trends Microbiol.* (2004) 12:129–34. 10.1016/j.tim.2004.01.001 15001189

[26] Backhed F Ley RE Sonnenburg JL Peterson DA Gordon JI. Host-bacterial mutualism in the human intestine. *Science.* (2005) 307:1915–20. 10.1126/science.1104816 15790844

[27] Hooper LV Littman DR Macpherson AJ. Interactions between the microbiota and the immune system. *Science.* (2012) 336:1268–73. 10.1126/science.1223490 22674334 PMC4420145

[28] Braniste V Al-Asmakh M Kowal C Anuar F Abbaspour A Toth M The gut microbiota influences blood-brain barrier permeability in mice. *Sci Trans Med.* (2014) 6:263ra158. 10.1126/scitranslmed.3009759 25411471 PMC4396848

[29] Diaz Heijtz R Wang S Anuar F Qian Y Bjorkholm B Samuelsson A Normal gut microbiota modulates brain development and behavior. *Proc Natl Acad Sci USA.* (2011) 108:3047–52. 10.1073/pnas.1010529108 21282636 PMC3041077

[30] Maamar E Ferjani S Jendoubi A Hammami S Hamzaoui Z Mayonnove-Coulange L High prevalence of gut microbiota colonization with broad-spectrum cephalosporin resistant *Enterobacteriaceae* in a tunisian intensive care unit. *Front Microbiol.* (2016) 7:1859. 10.3389/fmicb.2016.01859 27965626 PMC5126703

[31] Xu J Liang R Zhang W Tian K Li J Chen X Faecalibacterium prausnitzii-derived microbial anti-inflammatory molecule regulates intestinal integrity in diabetes mellitus mice via modulating tight junction protein expression. *J Diabet.* (2020) 12:224–36. 10.1111/1753-0407.12986 31503404 PMC7064962

[32] Vollaard EJ Clasener HA. Colonization resistance. *Antim Agents Chemother.* (1994) 38:409–14. 10.1128/aac.38.3.409 8203832 PMC284472

[33] Koh GY Kane AV Wu X Crott JW. Parabacteroides distasonis attenuates tumorigenesis, modulates inflammatory markers, and promotes intestinal barrier integrity in azoxymethane-treated A/J mice. *Carcinogenesis.* (2020) 41:909–17. 10.1093/carcin/bgaa018 32115637

[34] Zhai Q Feng S Arjan N Chen W. A next generation probiotic, akkermansia muciniphila. *Crit Rev Food Sci Nutr.* (2019) 59:3227–36. 10.1080/10408398.2018.1517725 30373382

[35] Lopes MP Cruz AA Xavier MT Stocker A Carvalho-Filho P Miranda PM Prevotella intermedia and periodontitis are associated with severe asthma. *J Periodontol.* (2020) 91:46–54. 10.1002/JPER.19-0065 31342509

[36] Kimizu T Takahashi Y Oboshi T Horino A Omatsu H Koike T Chronic dysfunction of blood-brain barrier in patients with post-encephalitic/encephalopathic epilepsy. *Seizure.* (2018) 63:85–90. 10.1016/j.seizure.2018.11.005 30465969

[37] Haruwaka K Ikegami A Tachibana Y Ohno N Konishi H Hashimoto A Dual microglia effects on blood brain barrier permeability induced by systemic inflammation. *Nat Commun.* (2019) 10:5816. 10.1038/s41467-019-13812-z 31862977 PMC6925219

[38] Canfora EE Jocken JW Blaak EE. Short-chain fatty acids in control of body weight and insulin sensitivity. *Nat Rev Endocrinol.* (2015) 11:577–91. 10.1038/nrendo.2015.128 26260141

[39] Macfarlane S Macfarlane GT. Regulation of short-chain fatty acid production. *Proc Nutr Soc.* (2003) 62:67–72. 10.1079/PNS2002207 12740060

[40] Louis P Flint HJ. Diversity, metabolism and microbial ecology of butyrate-producing bacteria from the human large intestine. *Fems Microbiol Lett.* (2009) 294:1–8. 10.1111/j.1574-6968.2009.01514.x 19222573

[41] Zhang J Song L Wang Y Liu C Zhang L Zhu S Beneficial effect of butyrate-producing Lachnospiraceae on stress-induced visceral hypersensitivity in rats. *J Gastroenterol Hepatol.* (2019) 34:1368–76. 10.1111/jgh.14536 30402954 PMC7379616

[42] Miller TL Wolin MJ. Bioconversion of cellulose to acetate with pure cultures of ruminococcus albus and a hydrogen-using acetogen. *Appl Environ Microbiol.* (1995) 61:3832–5. 10.1128/AEM.61.11.3832-3835.1995 16535158 PMC1388594

[43] Zhang M Zhou L Wang Y Dorfman RG Tang D Xu L Faecalibacterium prausnitzii produces butyrate to decrease c-Myc-related metabolism and Th17 differentiation by inhibiting histone deacetylase 3. *Int Immunol.* (2019) 31:499–514. 10.1093/intimm/dxz022 30809639

[44] Pryde SE Duncan SH Hold GL Stewart CS Flint HJ. The microbiology of butyrate formation in the human colon. *Fems Microbiol Lett.* (2002) 217:133–9. 10.1111/j.1574-6968.2002.tb11467.x 12480096

[45] De Vadder F Kovatcheva-Datchary P Goncalves D Vinera J Zitoun C Duchampt A Microbiota-generated metabolites promote metabolic benefits via gut-brain neural circuits. *Cell.* (2014) 156:84–96. 10.1016/j.cell.2013.12.016 24412651

[46] Macfabe DF. Short-chain fatty acid fermentation products of the gut microbiome: implications in autism spectrum disorders. *Microb Ecol Health Dis.* (2012) 23:19260. 10.3402/mehd.v23i0.19260 23990817 PMC3747729

[47] Kim HJ Leeds P Chuang DM. The HDAC inhibitor, sodium butyrate, stimulates neurogenesis in the ischemic brain. *J Neurochem.* (2009) 110:1226–40. 10.1111/j.1471-4159.2009.06212.x 19549282 PMC2726719

[48] Chen PS Wang CC Bortner CD Peng GS Wu X Pang H Valproic acid and other histone deacetylase inhibitors induce microglial apoptosis and attenuate lipopolysaccharide-induced dopaminergic neurotoxicity. *Neuroscience.* (2007) 149:203–12. 10.1016/j.neuroscience.2007.06.053 17850978 PMC2741413

[49] Kim HJ Rowe M Ren M Hong JS Chen PS Chuang DM. Histone deacetylase inhibitors exhibit anti-inflammatory and neuroprotective effects in a rat permanent ischemic model of stroke: multiple mechanisms of action. *J Pharmacol Exp Therap.* (2007) 321:892–901. 10.1124/jpet.107.120188 17371805

[50] Wang Z Leng Y Tsai LK Leeds P Chuang DM. Valproic acid attenuates blood-brain barrier disruption in a rat model of transient focal cerebral ischemia: the roles of HDAC and MMP-9 inhibition. *J Cereb Blood Flow Metab.* (2011) 31:52–7. 10.1038/jcbfm.2010.195 20978517 PMC3049473

[51] Park MJ Sohrabji F. The histone deacetylase inhibitor, sodium butyrate, exhibits neuroprotective effects for ischemic stroke in middle-aged female rats. *J Neuroinflammation.* (2016) 13:300. 10.1186/s12974-016-0765-6 27905989 PMC5131416

[52] Kim MH Kang SG Park JH Yanagisawa M Kim CH. Short-chain fatty acids activate GPR41 and GPR43 on intestinal epithelial cells to promote inflammatory responses in mice. *Gastroenterology.* (2013) 145:396–406. 10.1053/j.gastro.2013.04.056 23665276

[53] Pan X Fang X Wang F Li H Niu W Liang W Butyrate ameliorates caerulein-induced acute pancreatitis and associated intestinal injury by tissue-specific mechanisms. *Br J Pharmacol.* (2019) 176:4446–61. 10.1111/bph.14806 31347703 PMC6932943

[54] Chen G Ran X Li B Li Y He D Huang B Sodium butyrate inhibits inflammation and maintains epithelium barrier integrity in a TNBS-induced inflammatory bowel disease mice model. *EBioMedicine.* (2018) 30:317–25. 10.1016/j.ebiom.2018.03.030 29627390 PMC5952406

[55] Kamath PS Phillips SF Zinsmeister AR. Short-chain fatty acids stimulate ileal motility in humans. *Gastroenterology.* (1988) 95:1496–502. 10.1016/s0016-5085(88)80068-4 3181675

[56] Richardson A Delbridge AT Brown NJ Rumsey RD Read NW. Short chain fatty acids in the terminal ileum accelerate stomach to caecum transit time in the rat. *Gut.* (1991) 32:266–9. 10.1136/gut.32.3.266 2013421 PMC1378831

[57] Suzuki T Yoshida S Hara H. Physiological concentrations of short-chain fatty acids immediately suppress colonic epithelial permeability. *Br J Nutr.* (2008) 100:297–305. 10.1017/S0007114508888733 18346306

[58] Riviere A Selak M Lantin D Leroy F De Vuyst L. Bifidobacteria and butyrate-producing colon bacteria: importance and strategies for their stimulation in the human gut. *Front Microbiol.* (2016) 7:979. 10.3389/fmicb.2016.00979 27446020 PMC4923077

[59] Cani PD Possemiers S Van De Wiele T Guiot Y Everard A Rottier O Changes in gut microbiota control inflammation in obese mice through a mechanism involving GLP-2-driven improvement of gut permeability. *Gut.* (2009) 58:1091–103. 10.1136/gut.2008.165886 19240062 PMC2702831

[60] Al-Asmakh M Stukenborg JB Reda A Anuar F Strand ML Hedin L The gut microbiota and developmental programming of the testis in mice. *PLoS One.* (2014) 9:e103809. 10.1371/journal.pone.0103809 25118984 PMC4132106

[61] Velasquez-Manoff M. Gut microbiome: the peacekeepers. *Nature.* (2015) 518:S3–11. 10.1038/518S3a 25715278

[62] Luyer MD Buurman WA Hadfoune M Speelmans G Knol J Jacobs JA Strain-specific effects of probiotics on gut barrier integrity following hemorrhagic shock. *Infect Immun.* (2005) 73:3686–92. 10.1128/IAI.73.6.3686-3692.2005 15908398 PMC1111872

[63] Corr SC Li Y Riedel CU O’toole PW Hill C Gahan CG. Bacteriocin production as a mechanism for the antiinfective activity of *Lactobacillus salivarius* UCC118. *Proc Natl Acad Sci USA.* (2007) 104:7617–21. 10.1073/pnas.0700440104 17456596 PMC1863472

[64] Tok D Ilkgul O Bengmark S Aydede H Erhan Y Taneli F Pretreatment with pro- and synbiotics reduces peritonitis-induced acute lung injury in rats. *J Trauma.* (2007) 62:880–5. 10.1097/01.ta.0000236019.00650.00 17426542

[65] Beale RJ Sherry T Lei K Campbell-Stephen L Mccook J Smith J Early enteral supplementation with key pharmaconutrients improves sequential organ failure assessment score in critically ill patients with sepsis: outcome of a randomized, controlled, double-blind trial. *Critical Care Med.* (2008) 36:131–44. 10.1097/01.CCM.0000297954.45251.A9 18007263

[66] Kanauchi O Mitsuyama K Andoh A Iwanaga T. Modulation of intestinal environment by prebiotic germinated barley foodstuff prevents chemo-induced colonic carcinogenesis in rats. *Oncol Rep.* (2008) 20:793–801. 10.3892/or_0000076 18813820

[67] Sugawara G Nagino M Nishio H Ebata T Takagi K Asahara T Perioperative synbiotic treatment to prevent postoperative infectious complications in biliary cancer surgery: a randomized controlled trial. *Ann Surgery.* (2006) 244:706–14. 10.1097/01.sla.0000219039.20924.88 17060763 PMC1856608

[68] Shimizu K Yamada T Ogura H Mohri T Kiguchi T Fujimi S Synbiotics modulate gut microbiota and reduce enteritis and ventilator-associated pneumonia in patients with sepsis: a randomized controlled trial. *Critical Care.* (2018) 22:239. 10.1186/s13054-018-2167-x 30261905 PMC6161427

[69] Mittal R Coopersmith CM. Redefining the gut as the motor of critical illness. *Trends Mol Med.* (2014) 20:214–23. 10.1016/j.molmed.2013.08.004 24055446 PMC3959633

[70] Marteau P Pochart P Dore J Bera-Maillet C Bernalier A Corthier G. Comparative study of bacterial groups within the human cecal and fecal microbiota. *Appl Environ Microbiol.* (2001) 67:4939–42. 10.1128/aem.67.10.4939-4942.2001 11571208 PMC93255

