# Supplementary material for: Dysbiosis of Gut Microbiota and Short-Chain Fatty Acids in Encephalitis: A Chinese Pilot Study
Source: Front Immunol. 2020 Aug 20;11:1994. doi: 10.3389/fimmu.2020.01994 (PMC7468513; doi:10.3389/fimmu.2020.01994)
Supplement: Supplementary file 1 [file Data_Sheet_1.docx]

| **SUPPLEMENTARY TABLE S1** \| Diagnostic criteria for encephalitis according to Granerod, J. |
| --- |
| Encephalopathy = (altered consciousness persisting for longer than 24 h, including lethargy, irritability or a change in personality or behaviour)  Encephalitis = encephalopathy AND evidence of CNS inflammation, demonstrated by at leas two or more of the following:  > fever or history of fever (≥38℃) during the presenting illness;  > seizures or focal neurological findings with evidence of brain parenchyma involvement;  > CSF pleocytosis (more than four white cells per μL)  > EEG findings indicative of encephalitis  > abnormal results of neuroimaging (CT or MRI) suggestive of encephalitis |
| CNS, central nervous system; CSF, cerebrospinal fluid; EEG, electroencephalography |

| **SUPPLEMENTARY TABLE S2** \| GC/MS Analytical method. | |
| --- | --- |
| Parameter | Value |
| GC/MS System | Agilent 7890B GC/5977B MSD |
| Column | HP-FFAP 30 m × 0.25 mm, 0.25 μm (p/n 19091F-433) |
| Column flow | 1.0 mL/min |
| Liner | Agilent liner, split, single taper, wool, deactivated (p/n 5183-4711) |
| Injection mode | Split (30:1) |
| Injection temperature | 230 °C |
| Oven temperature | 90°C (1.0min), 15°C/min to 120 °C (0.5min),  15℃/min to 150℃ (0.5min),  15℃/min to 180℃ (1.0min). |
| Transfer line temperature | 230 °C |
| MS mode | SIM |
| SIM ions m/Z | 43,45,60,73,74,88 |
| Ion source | 5977 Inert Ion source Assy (p/n G3870-67700) |
| Ion source temperature | 230 °C |
| Quad. Temperature | 150 °C |
| GC/MS Data Acquisition | MassHunter GC/MS Acquisition Ver B.07.05.2479 |
| GC/MS Data Quantitative Analysis | MassHunter Workstation Software/ Quantitative Analysis Ver B.08.00 |

| **SUPPLEMENTARY TABLE S3** \| Diversity and richness index results from alpha-diversity analyses in twenty-eight Encephalitis patients and healthy subjects. | | | | | |
| --- | --- | --- | --- | --- | --- |
| **Patient** | **Shannon** | **PD-whole tree** | **Chao1** | **Observed species** | **Simpson** |
| ENC01 | 5.28 | 46.04 | 958.15 | 610 | 0.82 |
| ENC02 | 4.57 | 38.72 | 804.24 | 478 | 0.85 |
| ENC03 | 5.04 | 32.46 | 634.68 | 369 | 0.91 |
| ENC04 | 5.72 | 37.04 | 800.52 | 490 | 0.94 |
| ENC05 | 5.07 | 42.46 | 1000.50 | 579 | 0.83 |
| ENC06 | 6.14 | 39.78 | 888.54 | 527 | 0.96 |
| ENC07 | 6.04 | 40.24 | 1007.07 | 552 | 0.96 |
| ENC08 | 5.25 | 41.38 | 975.25 | 544 | 0.90 |
| ENC09 | 6.20 | 44.37 | 956.89 | 600 | 0.95 |
| ENC10 | 5.27 | 32.92 | 902.17 | 448 | 0.93 |
| ENC11 | 5.62 | 33.32 | 862.06 | 420 | 0.95 |
| ENC12 | 5.10 | 37.46 | 896.40 | 464 | 0.92 |
| ENC13 | 6.93 | 46.75 | 1029.66 | 632 | 0.98 |
| ENC14 | 5.16 | 26.39 | 447.98 | 299 | 0.93 |
| ENC15 | 5.05 | 29.29 | 580.59 | 365 | 0.92 |
| ENC16 | 6.03 | 39.93 | 895.39 | 520 | 0.95 |
| ENC17 | 4.26 | 33.49 | 703.57 | 394 | 0.79 |
| ENC18 | 3.83 | 37.10 | 781.39 | 439 | 0.70 |
| ENC19 | 6.25 | 42.26 | 1037.51 | 565 | 0.95 |
| ENC20 | 2.80 | 31.33 | 724.34 | 350 | 0.49 |
| ENC21 | 5.06 | 30.65 | 674.75 | 379 | 0.91 |
| ENC22 | 6.09 | 42.21 | 828.16 | 493 | 0.96 |
| ENC23 | 5.22 | 35.16 | 698.16 | 444 | 0.94 |
| ENC24 | 6.21 | 38.00 | 798.32 | 491 | 0.97 |
| ENC25 | 6.87 | 45.65 | 974.11 | 596 | 0.97 |
| ENC26 | 4.27 | 33.29 | 697.07 | 405 | 0.79 |
| ENC27 | 5.11 | 31.79 | 653.20 | 393 | 0.90 |
| ENC28 | 6.20 | 36.50 | 870.63 | 476 | 0.97 |
| CON01 | 5.25 | 34.23 | 945.02 | 471 | 0.89 |
| CON02 | 5.55 | 32.78 | 883.76 | 473 | 0.92 |
| CON03 | 3.98 | 32.62 | 684.18 | 415 | 0.70 |
| CON04 | 3.61 | 22.77 | 441.57 | 260 | 0.73 |
| CON05 | 5.28 | 31.76 | 885.11 | 467 | 0.87 |
| CON06 | 6.49 | 41.80 | 1075.83 | 598 | 0.96 |
| CON07 | 6.67 | 43.32 | 1178.70 | 625 | 0.97 |
| CON08 | 6.91 | 38.47 | 961.82 | 588 | 0.98 |
| CON09 | 5.44 | 31.56 | 724.44 | 418 | 0.92 |
| CON10 | 6.95 | 43.75 | 1044.48 | 667 | 0.97 |
| CON11 | 7.17 | 44.73 | 1133.07 | 669 | 0.98 |
| CON12 | 4.92 | 34.00 | 743.40 | 448 | 0.90 |
| CON13 | 5.61 | 34.20 | 734.47 | 470 | 0.92 |
| CON14 | 4.77 | 30.74 | 697.16 | 409 | 0.84 |
| CON15 | 6.52 | 37.69 | 952.45 | 533 | 0.98 |
| CON16 | 6.27 | 43.06 | 1030.43 | 629 | 0.95 |
| CON17 | 5.70 | 32.36 | 811.76 | 450 | 0.94 |
| CON18 | 4.65 | 29.33 | 750.11 | 414 | 0.85 |
| CON19 | 6.49 | 38.71 | 1039.53 | 573 | 0.97 |
| CON20 | 5.99 | 39.81 | 932.52 | 594 | 0.93 |
| CON21 | 6.04 | 33.37 | 801.54 | 479 | 0.96 |
| CON22 | 6.31 | 39.93 | 932.53 | 576 | 0.96 |
| CON23 | 5.27 | 37.38 | 798.84 | 505 | 0.88 |
| CON24 | 5.14 | 35.12 | 827.86 | 499 | 0.85 |
| CON25 | 5.83 | 36.80 | 967.63 | 519 | 0.93 |
| CON26 | 6.68 | 37.90 | 902.04 | 539 | 0.97 |
| CON27 | 5.90 | 30.26 | 692.85 | 431 | 0.95 |
| CON28 | 5.80 | 35.77 | 812.21 | 476 | 0.95 |
| PD-whole tree, phylogenetic diversity–whole tree. | | | | | |
