# Supplementary material for: Dysbiosis of Gut Microbiota and Short-Chain Fatty Acids in Encephalitis: A Chinese Pilot Study
Source: Front Immunol. 2020 Aug 20;11:1994. doi: 10.3389/fimmu.2020.01994 (PMC7468513; doi:10.3389/fimmu.2020.01994)
Supplement: Supplementary file 2 [file Data_Sheet_2.pdf]

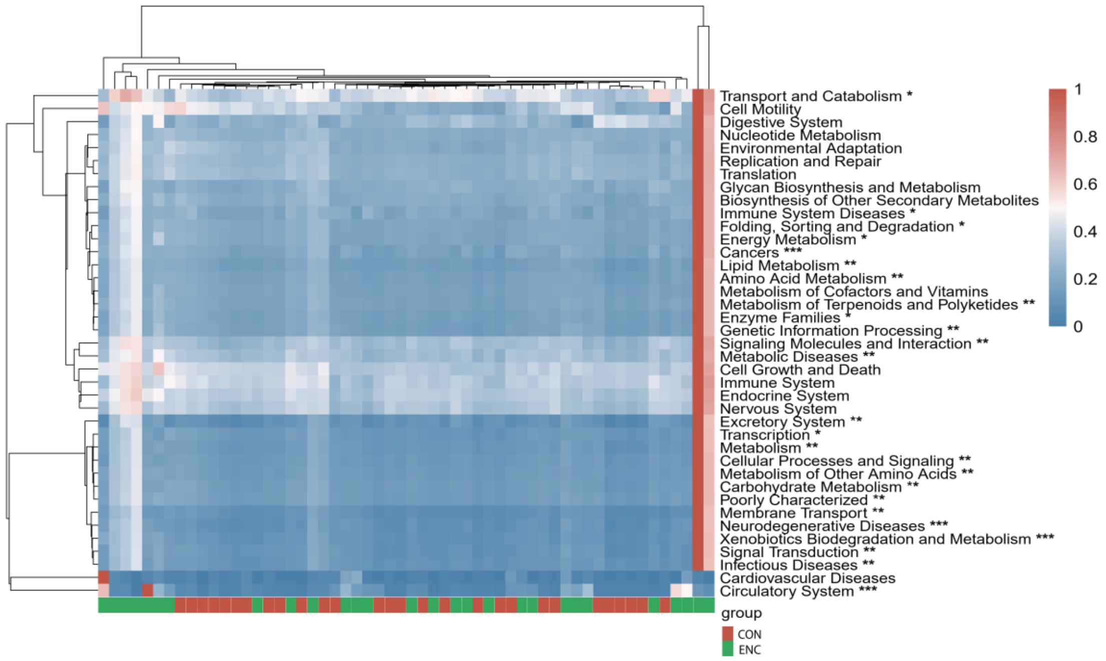

SUPPLEMENTARY FIGURE 1 | A comparison of KEGG predictive profiling by PICRUSt analysis. Among the 39 KEGG pathways, 25 pathways marked with "*", "**", or "***" were significantly upregulated in ENC group (*, p<0.05; **, p<0.01; ***, p<0.001). ENC, patients with encephalitis; CON, healthy subjects serving as controls.

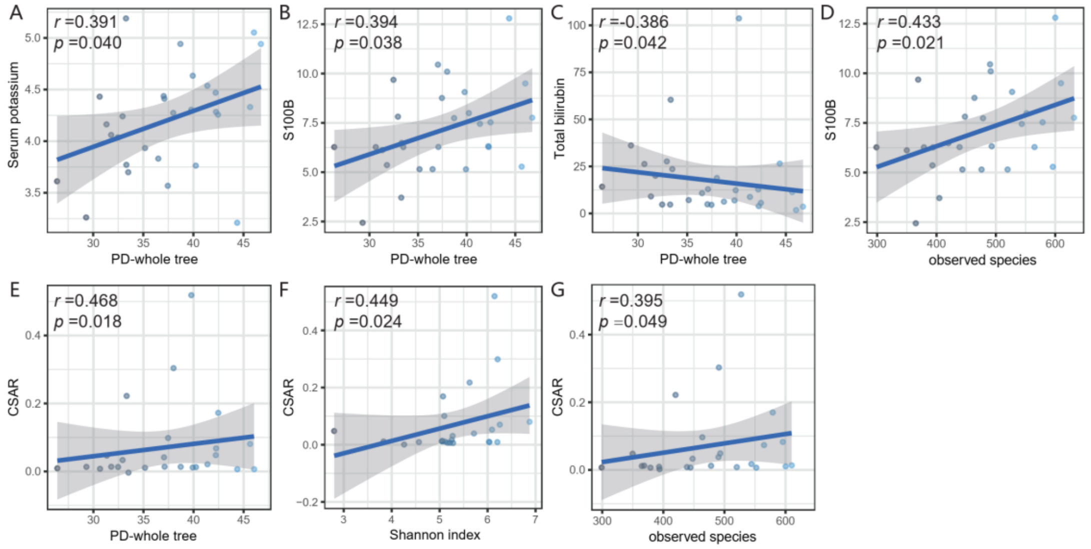

SUPPLEMENTARY FIGURE 2 | Spearman correlation analysis among several clinical parameters and microbial indexes. Correlations were found among PD-whole tree and serum potassium (A), S100B (B), total bilirubin (C), and the CSAR (E). A positive correlation was found between observed species and S100B (D). Positive correlations were found among the CSAR and PD-whole tree (E), Shannon index (F) and observed species (G). Statistical analyses were performed by Spearman's test. Significance was set at $p < 0.05$. PD-whole tree, phylogenetic diversity-whole tree; S100B, S100 calcium-binding protein B; CSAR, cerebrospinal fluid albumin-to-serum albumin ratio.

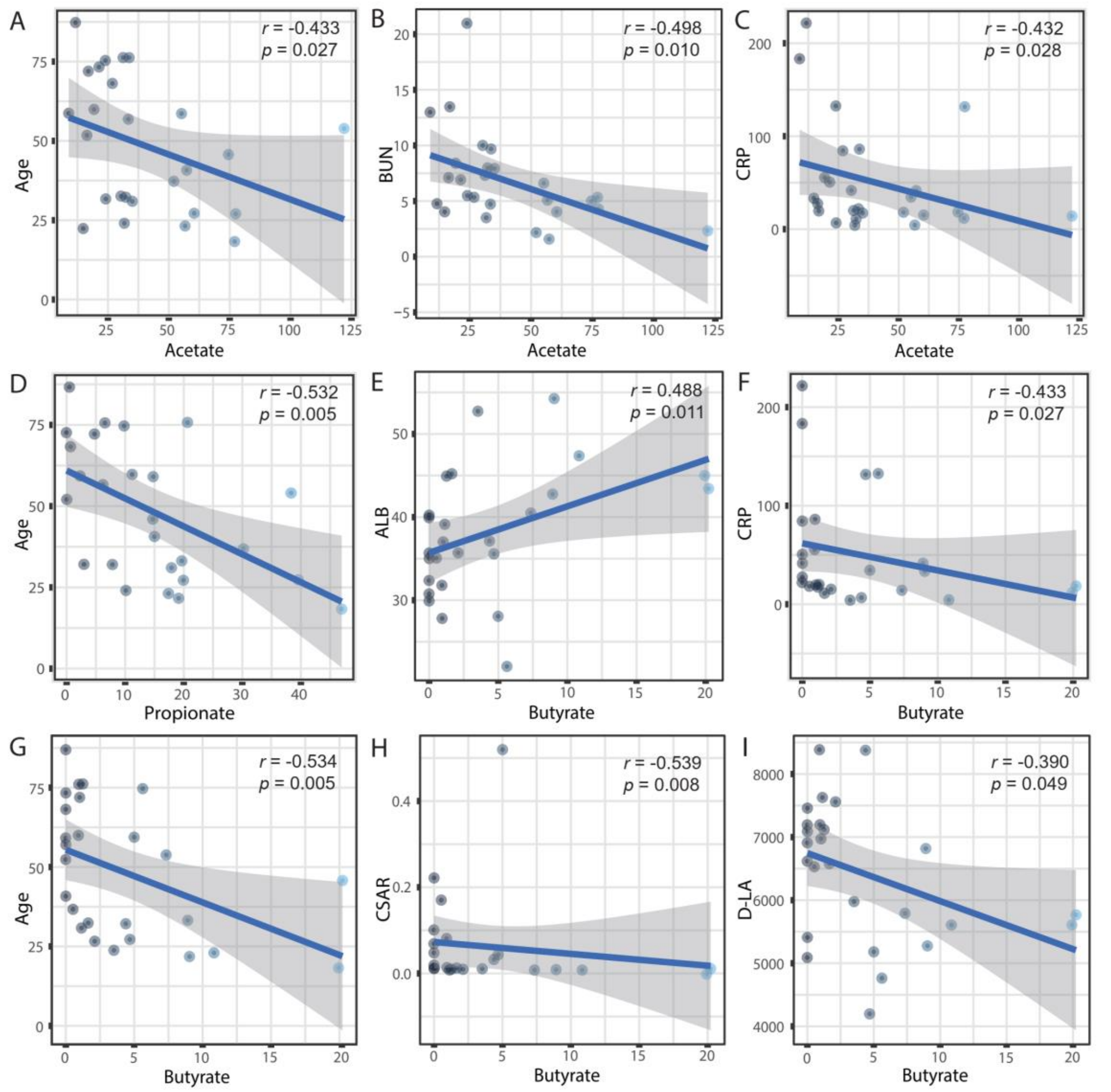

SUPPLEMENTARY FIGURE 3 | Spearman correlation analysis between blood parameters and fecal SCFAs. Negative correlations found among acetate and age (A), BUN (B) and CRP (C). Negative correlations found between propionate and age (D). Correlations found among butyrate and ALB (E), CRP (F), age (G), CSAR (H) and D-LA (I). Statistical analyses were performed by Spearman's test. Significance was set at $p < 0.05$. BUN, blood urea nitrogen; CRP, C-reactive protein; ALB, albumin; CSAR, cerebrospinal fluid albumin albumin-to-serum albumin ratio; D-LA, D-lactate.

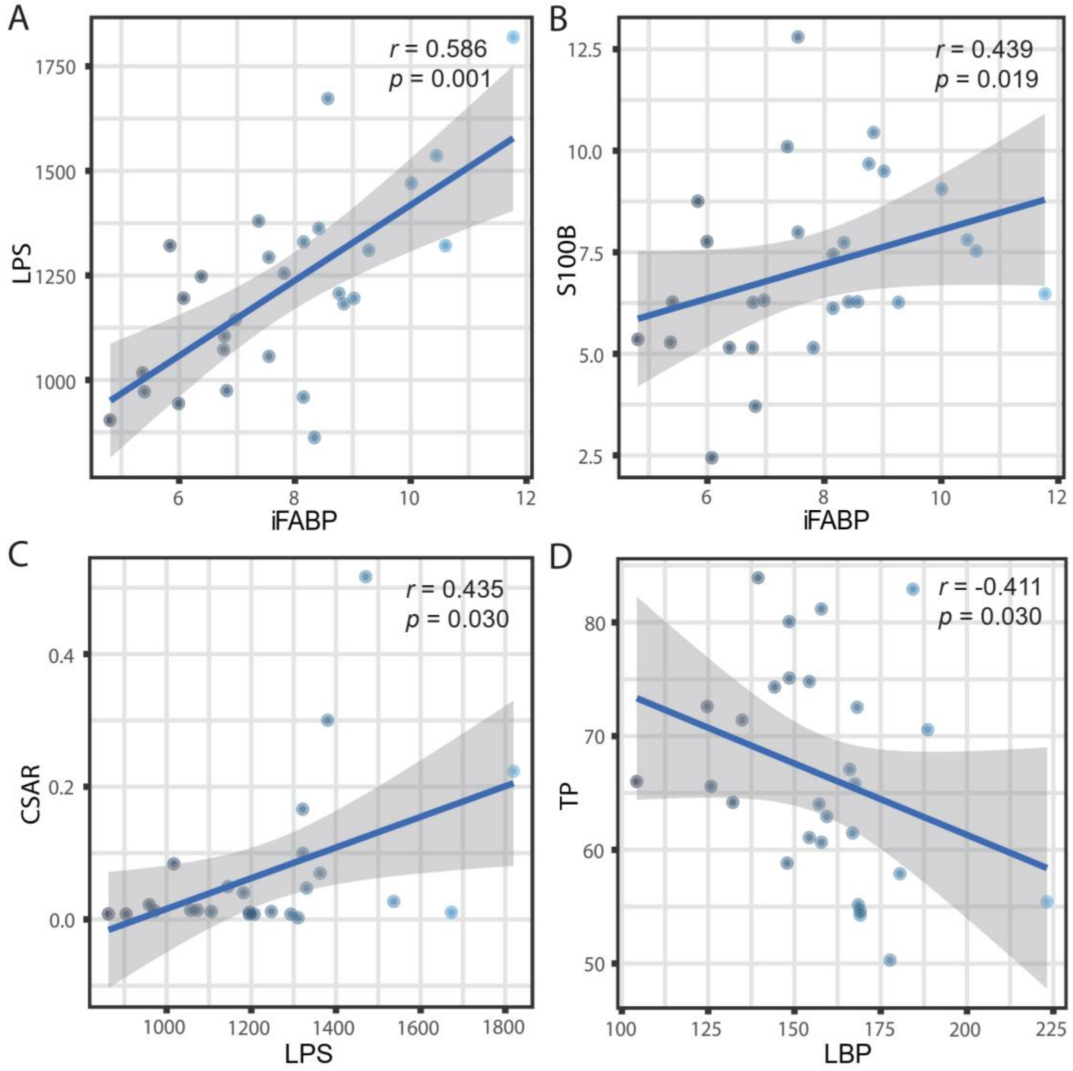

SUPPLEMENTARY FIGURE 4 | Spearman correlation analysis among selected blood parameters. Positive correlations were found among iFABP and LPS (A), S100B (B). Positive correlation were found between LPS and CSAR (C). Negative correlation were found between LBP and TP (D). Statistical analyses were performed by Spearman's test. Significance was set at $p < 0.05$. iFABP, intestinal fatty acid-binding protein; LPS, lipopolysaccharide; LBP, lipopolysaccharide-binding protein; S100B, S100 calcium-binding protein B; CSAR, cerebrospinal fluid albumin-to-serum albumin ratio; TP, total protein.
